# Optimized parameters for effective SARS-CoV-2 inactivation using UVC-LED at 275 nm

**DOI:** 10.1038/s41598-022-20813-4

**Published:** 2022-10-05

**Authors:** Cheulkyu Lee, Ki Hoon Park, Minjee Kim, Young Bong Kim

**Affiliations:** 1 Transportation Environmental Research Team, Korea Railroad Research Institute, 176, Cheoldobangmulgwan-ro, Uiwang-si, Gyeonggi-do, Republic of Korea; 2 Department of Biomedical Science and Engineering, KU Convergence Science and Technology Institute, Konkuk University, Seoul, 05029 Republic of Korea

**Keywords:** Antimicrobials, Optics and photonics

## Abstract

The spread of SARS-CoV-2 infections and the severity of the coronavirus disease of 2019 (COVID-19) pandemic have resulted in the rapid development of medications, vaccines, and countermeasures to reduce viral transmission. Although new treatment strategies for preventing SARS-CoV-2 infection are available, viral mutations remain a serious threat to the healthcare community. Hence, medical devices equipped with virus-eradication features are needed to prevent viral transmission. UV-LEDs are gaining popularity in the medical field, utilizing the most germicidal UVC spectrum, which acts through photoproduct formation. Herein, we developed a portable and rechargeable medical device that can disinfect SARS-CoV-2 in less than 10 s by 99.9%, lasting 6 h. Using this device, we investigated the antiviral effect of UVC-LED (275 nm) against SARS-CoV-2 as a function of irradiation distance and exposure time. Irradiation distance of 10–20 cm, < 10 s exposure time, and UV doses of > 10 mJ/cm^2^ were determined optimal for SARS-CoV-2 elimination (≥ 99.99% viral reduction). The UVC-LED systems have advantages such as fast-stabilizing intensity and insensitivity to temperature, and may contribute to developing medical devices capable of containing SARS-CoV-2 infection. By demonstrating SARS-CoV-2 inactivation with very short-term UVC-LED irradiation, our study may suggest guidelines for securing a safer medical environment.

## Introduction

The coronavirus disease of 2019 (COVID-19) pandemic has spread worldwide since its initial outbreak in 2019, causing serious morbidity and mortality. It is caused by SARS-CoV-2 (severe acute respiratory syndrome coronavirus 2), a highly contagious virus, detected mainly in specimens from the respiratory tract and nasopharyngeal sites in COVID-19 patients^1^. Reports indicate transmission between humans within 2–10 days, showing that the virus spreads through direct contact, such as contaminated hands and surfaces, and via airborne routes^2^. Under environmental conditions, SARS-CoV-2 remains viable in aerosols for up to 3 h and is more stable on plastic and stainless steel (up to 72 h) than on copper (4 h) and cardboard (24 h)^3^. Exposure to contaminated environmental materials can be prevented by many control techniques, including heat sterilization, chemical disinfection, filtration disinfecting surfaces, and ultraviolet (UV) irradiation^4^. The possible material damage caused by heat sterilization and toxicity of chemical disinfectants, and the shortage of filters in the market pose a major challenge throughout the pandemic, generating an alarming demand of more sustainable disinfection systems^4^. Given the rapid transmission of the virus, it is important to develop sustainable measures and technologies that can inactivate the virus and limit transmission.

The global UVC (ultraviolet-C) market growth has been positively impacted by the COVID-19 outbreak. During the pandemic, UV air and surface disinfection has attracted attention to UV devices and many products became available on the market^4^. Various public places with different levels of contaminated air and environmental materials started using UV surface disinfection systems^4^. UV rays are classified into three basic types according to wavelength: UVA (320–400 nm), UVB (280–320 nm), and UVC (100–280 nm)^5^. Various research centers and laboratories are developing UVC-based products to prevent the spread of infection. UV light-emitting diodes (UV-LEDs) are mercury-free devices that can be used for on-demand operations^6^. While mercury lamps emit light only at a particular wavelength, UV-LEDs are capable of emitting light at multiple individual wavelengths^5^. As a public health and environmental safety measure, the United Nations Environment Programme (UNEP) banned mercury-containing products in 2013 and beginning in 2020, low-pressure mercury lamps were to be replaced with new UV-emitting sources^7^. UV irradiation is an emerging antimicrobial approach owing to its flexibility, availability, and easy control of radiation patterns^8^. Medical devices equipped with UV-LEDs are now gaining popularity in medical fields, with UVC, which acts through the formation of photoproducts, considered the most effective germicidal region within the UV spectrum^9^. Additionally, a recent study reported that the UVC-LED intensity was not affected by temperature changes or warm-up time^10^. Further, UVC-LED inactivates pathogens through several mechanisms, including nucleic acid or protein damage and producing oxygen radicals^11,12^. A recent study reported that irradiation with UVC-LED at a wavelength of 280 ± 5 nm rapidly inactivated SARS-COV-2 isolated from a COVID-19 patient^9^. Furthermore, another study reported the elimination of SARS-COV-2 upon treatment with high temperature (> 56 °C) and UVC irradiation (100–280 nm)^13^. Various technologies to disinfect COVID-19 employing UV include photoelectrochemical oxidation (PECO) technology used in developing an air purifier, wherein UV-A light was utilized to activate a catalyst in the nanoparticle-covered filter to oxidize air contaminants^14^. In accordance with these findings, we developed a portable and rechargeable medical device for SARS-CoV-2 disinfection, which can be utilized to sterilize hard-to-reach areas or surfaces that will stain or otherwise react upon contact with cleaning chemicals. In the current study, we demonstrate exposure time- and distance-dependent reductions in SARS-COV-2 by UVC and aim to optimize and validate the performance of the developed UVC-LED device.

## Methods

### UVC-LED irradiation system

A portable UVC device, manufactured by the Korea Railway Research Institute (KRRI), containing a 1000 mW LED module was used in the current study. The module also contained a cooling system and a human detection sensor, which were discarded after use to prevent contamination risks. UVC-exposure experiments were conducted using a UV-LED system with selected LEDs obtained from the Korea Institute of Lighting and ICT (Bucheon, Korea). The UV spectra of the UV-LED wavelengths used in this study were measured using an IDR300 Photobiological Safety Spectroradiometer (Bentham, Reading, UK).

### Virus irradiation with UVC-LED

The SARS-CoV-2 resource (NCCP43326) utilized in this study was procured from the National Culture Collection for Pathogens of the Korea Center for Disease Control and Prevention. VeroE6 cells (African green monkey kidney cell line) were purchased from the Korean Cell Line Bank (Seoul, Korea). For these experiments, 100 μL of the viral suspension with a titer of 3.16 × 10^6^ TCID_50_ (50% tissue culture infective dose)/mL was placed in a petri dish and covered with a quartz coverslip. The UVC-LED irradiance produced for the virus eradication was measured at different heights (10, 20, 30, and 50 cm) for different times (2–60 s). After UV exposure, the virus was collected and serially diluted tenfold and infected into the Vero-E6 cells. Infected cells were incubated for 3 d at 37 °C in a humidified 5% CO_2_ incubator, following which the cytotoxic effects were evaluated by staining with a crystal violet solution.

### Verification of viral titer reduction as a function of UV exposure time and distance

The Vero E6 cells infected with the post-irradiated virus were stained, and the TCID_50_ was calculated using the Spearman–Karber method. The viral titers and reduction rates were determined according to the exposure time and distance between the UV radiation device and virus-infected cells. Virus reduction was calculated according to the following equation:

1 $$ \frac{{{1}00 - {\text{Viral titer post - irradiation }}({\text{TCID}}_{{{5}0}} /{\text{ml}}) \times {1}00}}{{{\text{Initial viral titer}}({\text{TCID}}_{{{5}0}} /{\text{ml}})}} $$

### Verification of viral titer reduction according to UVC radiation dose

The UV dose was estimated by calculating UV irradiance based on exposure time (s) and distance (cm) between the UV-LED and virus surface. In this study, a device with a luminous intensity of 1000 mW was used, but considering the experimental loss, a value of 800 mW was used to calculate the UV dose according to Eq. (2):

2 $$ {\text{UV dose }}({\text{mJ}}/{\text{cm}}^{{2}} ) = {\text{UV irradiance }}({\text{mW}}/{\text{cm}}^{{2}} ) \times {\text{time }}\left( {\text{s}} \right) $$

The distance between the UVC-LED device and the plated virus was set at 10, 20, 30, and 50 cm, and exposure times of 2, 4, 5, 10, 20, 30, 40, 50, and 60 s were used. The plated virus was covered with a quartz coverslip for even UV exposure. Following exposure, the virus was harvested by washing the quartz coverslip with complete media, then serially diluted tenfold, and used to infect Vero-E6 cells. After incubating for 3 days, viral titer reduction was measured by staining cells with a crystal violet solution.

### Statistical analysis

All measures of variance are presented as the standard error of the mean (SEM). Correlations of efficacy with irradiation distance and time and relationship to UV dose were analyzed using two-way analysis of variance (ANOVA) with a Tukey’s post-hoc test using Prism8 (GraphPad Software, San Diego, CA, USA).

## Results

### UVC-LED irradiance as a function of wavelength

**Figure 1:**
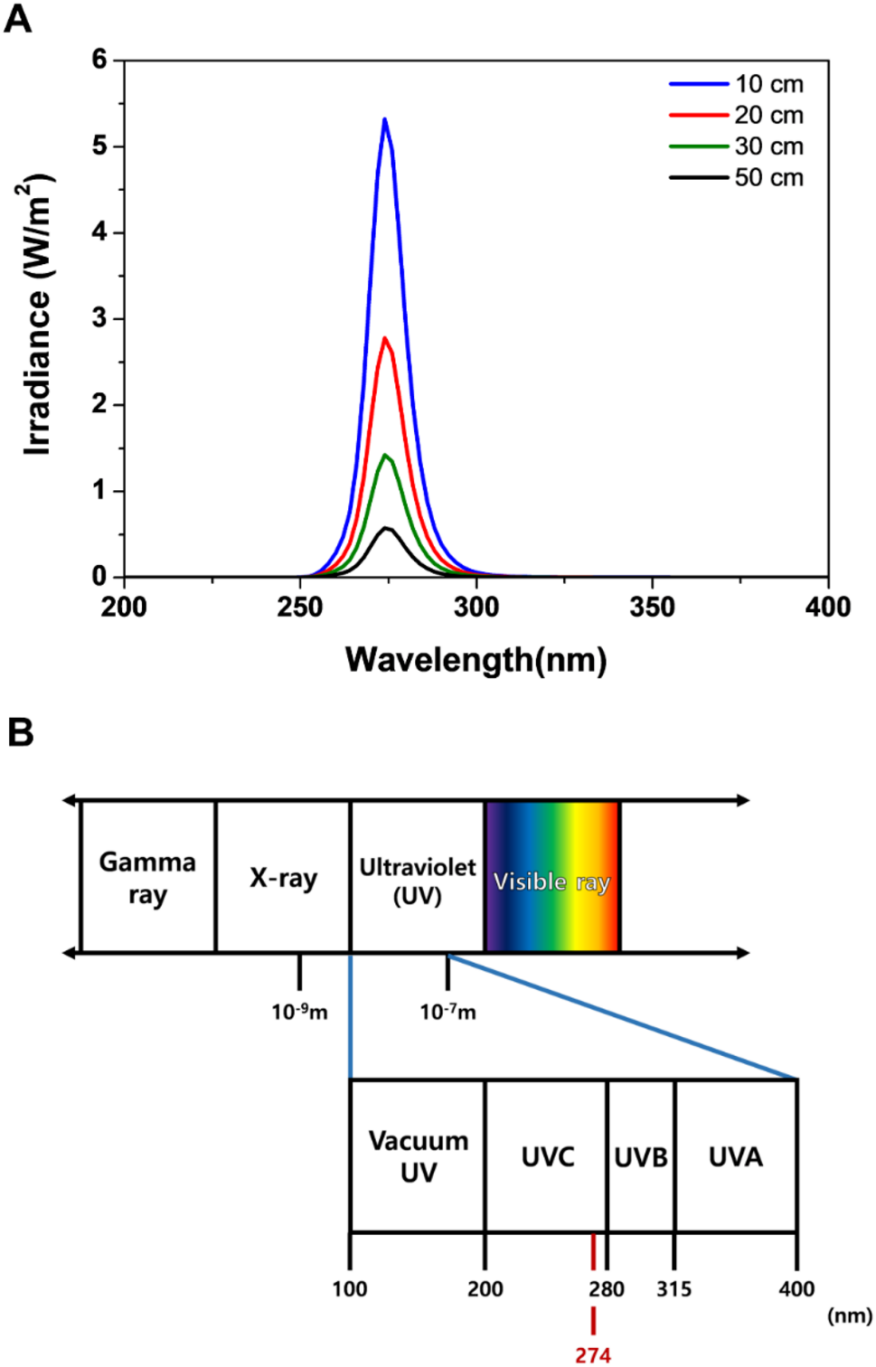
UV-LED irradiance as a function of wavelength. UV light corresponds to the area of light with wavelengths between 100 and 400 nm; a wavelength of 275 nm showed the highest measured irradiance in our study.

The UVC-LED irradiance generated for virus eradication was measured at heights of 10, 20, 30, and 50 cm. We measured irradiance at a wavelength of 275 nm and confirmed that the intensity of the 275 nm peak gradually decreased at the sample as the distance from the source increased (Fig. 1). Accordingly, we assessed viral eradication at various irradiation time points and distances.

### Viral reduction as a function of UVC exposure time and distance

**Figure 2:**
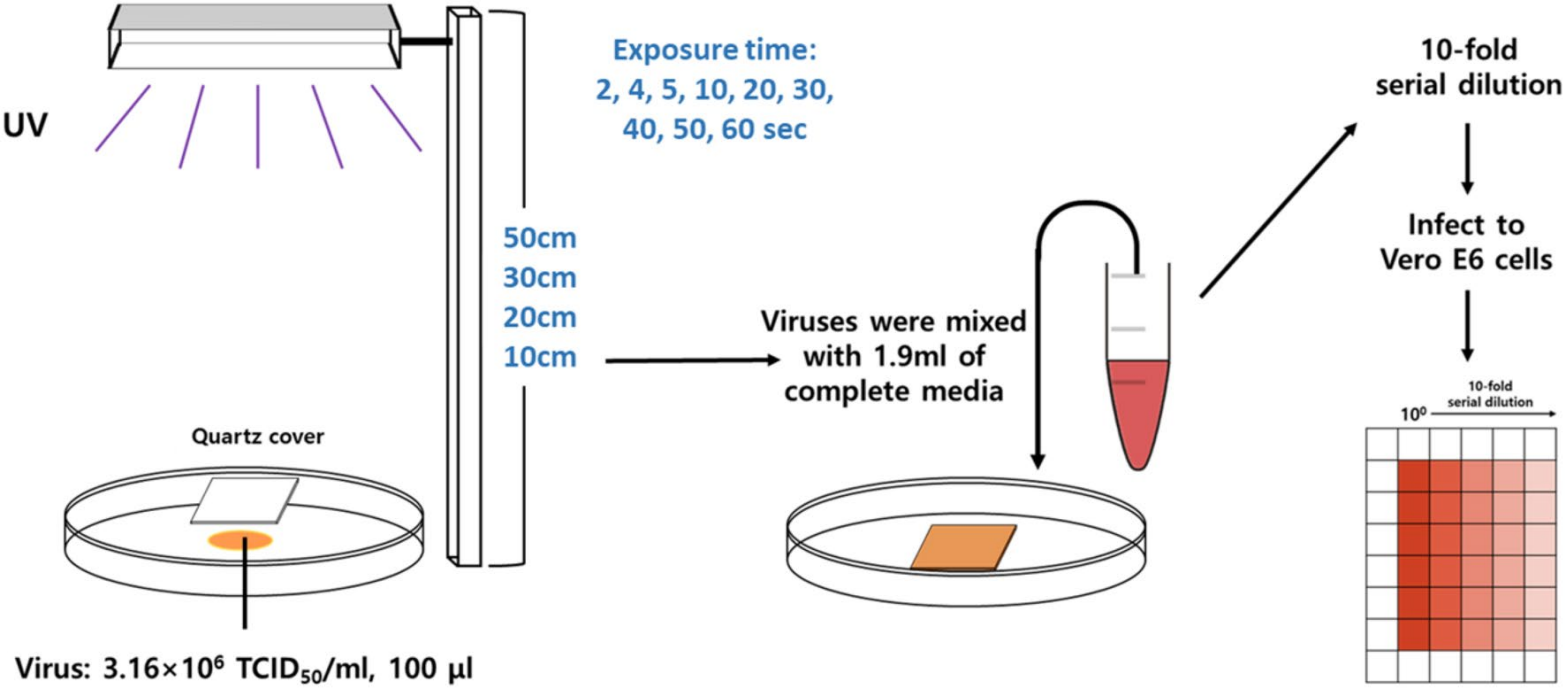
Schematic representation of UV irradiation test against SARS-CoV-2. The virus was exposed to ultraviolet C (UVC) at distances of 10, 20, 30, and 50 cm. The virus treated under each condition was serially diluted and infected into Vero E6 cells.

The distance between UVC-LED and plated virus (3.16 × 10^4^ TCID_50_/mL, 100 µL) was fixed, and the exposure time was varied. The virus was covered and exposed to UVC, after which the virus was harvested for infection into the Vero-E6 cells (Fig. 2).

**Figure 3:**
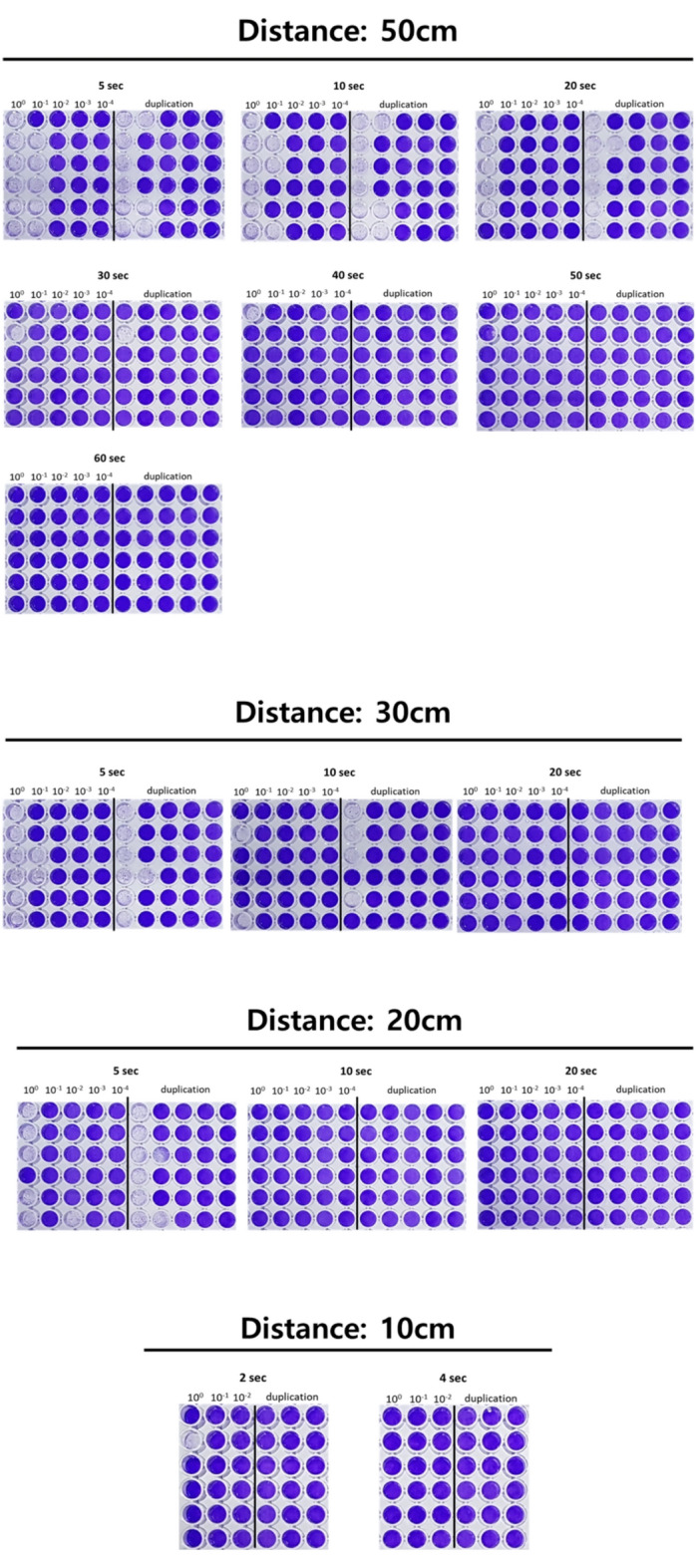
Crystal violet staining of Vero E6 cells infected with UV-irradiated SARS-CoV-2. Vero E6 cells were infected with UV-irradiated virus and incubated for 3 days. Cells were then stained with a crystal violet solution.

Post-irradiation, the viral reduction was measured as a function of varying UVC exposure time and distance by determining the viral titer (Fig. 3). After incubating for 3 days, cell death induced by virus infection was assessed by staining cells with a crystal violet solution. We observed that the viability of cells infected with UVC-irradiated virus gradually decreased with increasing UVC exposure time and a shorter distance between the virus and UVC-LED.

**Figure 4:**
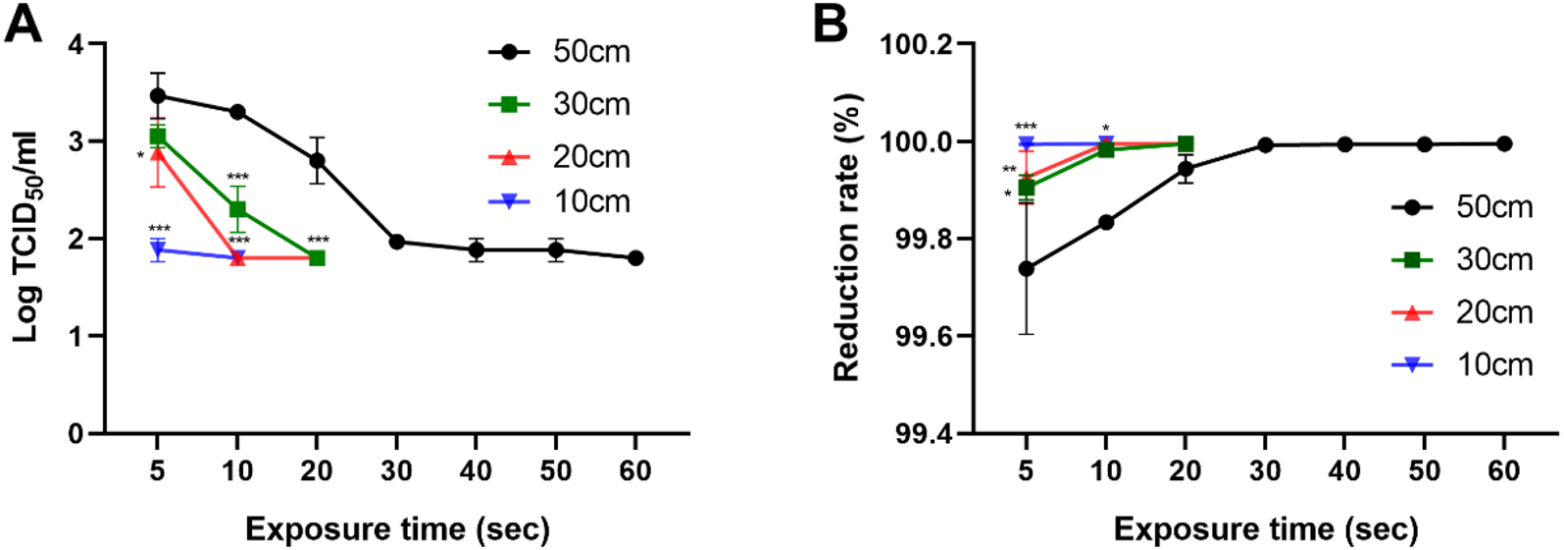
Verification of SARS-CoV-2 reduction as a function of ultraviolet C (UVC) exposure time and distance. After staining Vero E6 cells with crystal violet, 50% tissue culture infective dose (TCID_50_) was calculated using the Spearman–Karber method. (**A**) Determination of viral titer at variable radiation exposure time and distance between the UV light-emitting diode (UV-LED) and plated virus. (**B**) Determination of viral reduction rate over time at varying UV irradiation distance (*p ≤ 0.05, **p ≤ 0.005, ***p ≤ 0.0005 vs. 50 cm condition).

After staining the infected Vero E6 cells, TCID_50_ was calculated using the Spearman-Karber method (Fig. 4.). At 50 cm, the viral titers were calculated to be 3.2 × 10^3^, 2.0 × 10^3^, 6.8 × 10^2^, 9.3 × 10^1^, 7.8 × 10^1^, 7.8 × 10^1^, and 6.3 × 10^1^ TCID_50_/mL at irradiation times of 5, 10, 20, 30, 40, 50, and 60 s, respectively, yielding the corresponding log TCID_50_/mL values of 3.468, 3.301, 2.801, 1.968, 1.884, 1.884, and 1.801. From these values, a viral reduction of ≥ 99.99% was calculated compared with the unirradiated viral samples (3.16 × 10^6^ TCID_50_/mL, 6.500 log TCID_50_/mL) at irradiation times of > 30 s and a distance of 50 cm. At 30 cm, the viral titers were calculated to be 1.1 × 10^3^ TCID_50_/mL (3.031 log TCID_50_/mL), 2.2 × 10^2^ TCID_50_/mL (2.301 log TCID_50_/mL), and 6.3 × 10^1^ TCID_50_/mL (1.801 log TCID_50_/mL) at irradiation times of 5, 10, and 20 s, respectively. At 20 cm, viral titers were calculated as 8.96 × 10^2^ TCID_50_/mL (2.884 log TCID_50_/mL), 6.32 × 10^1^ TCID_50_/mL (1.801 log TCID_50_/mL), and 6.32 × 10^1^ TCID_50_/mL (1.801 log TCID_50_/mL) at irradiation times of 5, 10, and 20 s, respectively. Collectively, these data confirm a viral reduction rate of > 99.99% at 30 cm/20 s, 20 cm/10 s, and 20 cm/20 s. At 10 cm, viral titers were 7.80 × 10^1^ TCID_50_/mL (1.884 log TCID_50_/mL) and 6.32 × 10^1^ TCID_50_/mL (1.801 log TCID_50_/mL) at irradiation times of 2 and 4 s, both translating to a viral reduction rate of > 99.99%.

### SARS-CoV-2 titer reduction as a function of UVC irradiation strength

UV doses at different distances and exposure times were calculated using Eq. (2) and a power of 800 mW rather than the actual 1000 mW to account for the experimental loss (Table 1). Four conditions (red font) resulted in a ≥ 99.99% viral reduction: 30 s UV at 50 cm, 20 s UV at 30 cm, 10 s UV at 20 cm, and 2 s UV at 10 cm.

**Table 1:** UV irradiation dose as a function of exposure time and distance.

Time (s)	Distance (cm)				
	10	20	30	50	
2	**16**	4	2	1	**UV dose (mJ/cm**^**2**^**)**
4	32	8	4	1	
5	40	10	4	2	
10	80	**20**	9	3	
15	120	30	13	4.8	
20	160	40	**18**	6	
30	200	50	27	**10**	
40	140	60	36	13	
50	180	70	44	16	
60	480	120	53	19	

Significant values are in bold.

**Figure 5:**
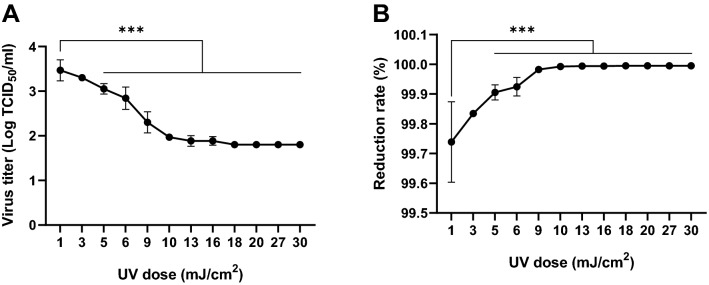
Verification of the SARS-CoV-2 titer reduction at different ultraviolet C (UVC) radiation doses. UV irradiance was deduced based on exposure time and distance between the UV light-emitting diode (UV-LED) and virus. (**A**) The variance of viral titer due to UV irradiation. (**B**) Evaluation of virus reduction rate by UV irradiation. The titer of non-irradiated virus was used as a negative control. ***p ≤ 0.0005.

Further, we verified the titer and viral reduction of SARS-CoV-2 as a function of UVC radiation (Fig. 5, Table 2). Interestingly, a common feature gleaned from these experiments is that the UV doses > 10 mJ/cm^2^ produced a 99.99% viral reduction. In case of the 20 cm/5 s condition, the calculated dose of 10 mJ/cm^2^, which is the same as determined for the 50 cm/30 s condition, showed ≥ 99.96% viral reduction, which is slightly lower than the 99.99% standard reduction. Therefore, we conclude that a UV dose of > 10 mJ/cm^2^ is required for a stable viral reduction of > 99.99%.

**Table 2:** UV irradiation dose as a function of Log_10_ reduction in infectious SARS-CoV-2 titer.

	UV dose (mJ/cm^2^)									Max^a^
	1	3	5	6	9	10	13	16	18	
Log_10_ reduction in infectious SARS-CoV-2 titer	2.61	2.78	3.03	3.16	3.78	4.11	4.20	4.20	≥ 4.28	4.28

^a^Maximum reduction value.

## Discussion

The spread of SARS-CoV-2 infection and the severity of the COVID-19 pandemic has raised worldwide concerns and has empowered the rapid development of medications, vaccines, and countermeasures to contain viral transmission. In this study, we investigated the antiviral effects of UVC-LED against SARS-CoV-2 at a wavelength of 275 nm at different distances and exposure times. The novelty of our study is that it presents the optimized SARS-CoV-2 deactivating conditions employing UVC-LED at a wavelength of 275 nm. We confirmed a viral reduction rate of ≥ 99.99% at 50 cm with irradiation for > 30 s, 30 cm with 20 s irradiation, 20 cm with 10 s irradiation, and 10 cm with 2 s irradiation. Collectively, these results reveal that a distance of 10–20 cm is ideal for rapid (< 10 s) viral eradication. According to our calculations, a UV dose of > 10 mJ/cm^2^ resulted in 99.99% reduction of the virus. Overall, we conclude that the distance of 10–20 cm between the UVC source and the virus contaminated surface, an exposure time of < 10 s, and a UV dose of > 10 mJ/cm^2^ are the ideal conditions for effective SARS-CoV-2 eradication.

Both community and healthcare settings are vulnerable to the spread of SARS-CoV-2, and the stability of SARS-CoV-2 will likely be a threat in both environments^3^. Although various clinical trials and vaccines are currently available for the treatment and prevention of SARS-CoV-2, viral mutations remain a serious threat to our healthcare community. Hence, medical devices equipped with virus-eradication features are required to prevent viral transmission in healthcare environments.

Devices equipped with UV-LEDs are now gaining popularity in the medical fields^9^. Within the UV spectrum, UVC is considered to have the most powerful germicidal effects, inactivating various microorganisms such as viruses, bacteria, protozoa, and fungi, among others, via formation of the pyrimidine dimers in DNA and RNA^9,15^. Consecutively, pyrimidine dimers are considered to be photoproducts that disrupt DNA replication and transcription, leading to cell death^16^. Shin et al. reported effective inactivation of *Escherichia coli* O157:H7, *Salmonella typhimurium*, and *Listeria monocytogenes* on medium surfaces using UVC-LED at a wavelength of 275 nm, and in water systems at 278 nm under various conditions^10^. Additionally, another study comparing different spectra of UVA, UVB, and UVC against influenza virus revealed that UVB- and UVC-LED irradiation were highly effective in inactivating the virus^5^. By demonstrating SARS-CoV-2 inactivation with very short-term UVC-LED irradiation and determining the optimal irradiation distances and exposure times, our study suggests guidelines for securing a safer medical environment. Considering the advantages of UVC-LED, such as rapidly stabilizing intensity and insensitivity to temperature^10^, this system may contribute to the development of medical devices capable of preventing SARS-CoV-2 infection.

## Data Availability

The datasets generated during the current study are available from the corresponding author (kimera@konkuk.ac.kr) on reasonable request.
